# Identification and prioritization of barriers to the implementation of an asset management system in the healthcare sector using a Delphi-AHP approach

**DOI:** 10.1108/JHOM-12-2024-0513

**Published:** 2025-11-26

**Authors:** Damjan Maletič, Justyna Trojanowska, Mateja Lorber, Matjaž Maletič

**Affiliations:** Faculty of Organizational Sciences, University of Maribor, Kranj, Slovenia; Faculty of Mechanical Engineering, Poznan University of Technology, Poznań, Poland; Faculty of Health Sciences, University of Maribor, Maribor, Slovenia

**Keywords:** Asset management, ISO 55001, Barriers, Healthcare, Delphi, AHP

## Abstract

**Purpose:**

An effective asset management system (AMS) is essential for healthcare organizations looking to maximize value and performance while minimizing risk and cost. This study aims to identify and evaluate the barriers to AMS adoption and evaluate them from a healthcare perspective.

**Design/methodology/approach:**

The study is based on a combination of the Delphi method and the analytic hierarchy process (AHP) with 30 participants from various Slovenian healthcare organizations. Through iterative consensus and prioritization, the Delphi-AHP process resulted in 23 validated barriers, ranked according to their perceived importance for AMS implementation.

**Findings:**

The results identified key barriers to implementing AMS in healthcare organizations and categorized them into five dimensions: strategic, human resources, contextual, structural and procedural. The highest-ranked barriers were deficient leadership, a shortage of qualified personnel and workforce overload. This indicates that strategic alignment and organizational capacity are perceived as the most critical obstacles to adopting AMS.

**Originality/value:**

This study advances the existing literature by addressing a critical gap and providing deeper insight into the factors that impede the successful implementation of AMS in healthcare settings, a domain where empirical evidence remains limited.

## 1. Introduction

Healthcare organizations face many challenges. They must develop effective management and control systems (Ippolito *et al.*, 2022), respond to the expectations of different stakeholders, and create value beyond monetary considerations (Maguire and Murphy, 2023). They must also improve the quality of care and the overall care experience (Shepherd *et al.*, 2019). To this end, healthcare organizations are seeking to enhance their efficiency (IHI, 2005) and thereby increase value (Ahmed *et al.*, 2019; Ali and Dzandu, 2023).

Depending on the context, the term “asset management” can have different meanings. However, ISO 55000, an international standard, defines asset management as “the coordinated activity of an organization to realize value from assets” (ISO 55000, 2024). According to the Institute of Asset Management (IAM, 2015), organizations increasingly recognize asset management as a relevant discipline with significant potential to improve performance. Although asset management can be viewed from multiple perspectives, the underlying logic is straightforward: a systematic and structured approach to managing the lifecycle of assets from acquisition to operation, maintenance, and disposal (ISO 55000, 2024). Healthcare organizations must understand the importance of effectively managing assets throughout their lifecycle. This is true in healthcare facility management (Shohet and Lavy, 2004), healthcare investment decisions (Vassolo *et al.*, 2021), workforce competency management (Zafošnik *et al.*, 2024), management of equipment maintenance (Bahreini *et al.*, 2019), risk management, change management, and leadership (Plsek and Wilson, 2001), among others. Assets can be divided into two categories: physical (tangible) assets, such as medical equipment and facilities, and intangible assets, such as intellectual and financial assets.

For an organization, setting up an asset management system (AMS) is a strategic decision. Various frameworks can help organizations set up an appropriate AMS, including the international standard ISO 55001 (ISO 55001, 2024) and the GFMAM Asset Management Landscape (GFMAM, 2024). An AMS encompasses elements within and outside an organization, including stakeholders, external service providers, and the organization's processes, activities, and functions (ISO 55000, 2024). Integrating the entire asset lifecycle into a holistic framework can support this (El-Akruti *et al.*, 2013; Sandu *et al.*, 2023; Schuman and Brent, 2005).

Despite the availability of asset management frameworks and standards, many organizations have not yet successfully used AMS to achieve the desired results (Maletič *et al.*, 2023). Organizations that are beginning to implement AMS must have a solid grasp of their existing organizational context to develop effective implementation strategies (ISO 55000, 2024). When pursuing the goal of implementing an organization-wide management system, an organization must demonstrate leadership commitment, holding top management accountable for the effectiveness of the management system (Kohl, 2020). In other disciplines, such as quality management, barriers such as strategic alignment, internal communication, resource availability, knowledge level, cross-departmental collaboration, and resistance to change have been identified as potential AMS barriers (Rawshdeh *et al.*, 2024). Drawing from research on evidence-based management in healthcare systems (Shafaghat *et al.*, 2021), additional fundamental barriers can be identified and grouped into categories: external factors (e.g. lack of stakeholder support); contextual factors (e.g. lack of organizational commitment and poor culture); limited resources (e.g. insufficient infrastructure and staff); policies and procedures (e.g. lack of clear systems, programs, and training); and research capacity and data availability (e.g. lack of high-quality evidence). One problem of fully understanding the barriers to AMS adoption is that knowledge of these barriers tends to be fragmented, i.e., specific to a particular industry and/or context. Numerous studies have investigated the introduction of AMS in industrial sectors such as infrastructure (e.g. Beitelmal *et al.*, 2017), but comparatively few have examined healthcare. A key limitation of the existing literature is the assumption that the barriers to implementation are largely the same across all sectors, which overlooks crucial contextual differences. Healthcare organizations often struggle with strict regulations, complex processes due to critical patient care and limited budgets – particularly in publicly funded systems (Rouse and Serban, 2014). These factors present unique challenges, such as staff resistance to change (Amarantou *et al.*, 2018), limited investment capacity and infrastructure management capability (Nandy, 2025), and difficulties in measuring asset performance (Salem and Elwakil, 2021). In contrast, industry tends to focus on cost efficiency, and standardized asset governance structures with clearly defined hierarchies prevail (Diop *et al.*, 2021). Consequently, the barriers identified in industry may not fully reflect the complexities of the healthcare sector. This emphasizes the need for sector-specific investigations of AMS implementation barriers, such as the one conducted in this study.

### 1.1 Positioning of the study

Although there is a clear message that organizations can benefit from adopting asset management practices (Lima *et al.*, 2021), AMS is often integrated into organizations in a superficial rather than effective manner (Maletič *et al.*, 2023). Although corporate interest in implementing AMS is growing (Alsyouf *et al.*, 2021), the literature reveals a notable lack of empirical studies exploring the barriers to effective adoption. This gap is especially apparent in healthcare, where AMS principles have only recently begun to influence organizational practices. Previous studies have focused on specific phases of the asset lifecycle, such as planning (Nezamian and Burns, 2020), the operation and maintenance phase (Bahreini *et al.*, 2019) and the disposal and replacement phase (Wang *et al.*, 2024). These studies often overlook the need for comprehensive, organization-wide frameworks. Furthermore, while asset management in public utilities such as water and electricity is relatively mature (Alquraidi and Awad, 2024), the healthcare sector remains understudied despite its growing complexity and demand for efficiency (Dehe and Bamford, 2017). Although the literature emphasizes the performance outcomes of AMS initiatives, it pays insufficient attention to the underlying organizational and human factors that impede successful implementation. Barriers such as a lack of skilled personnel, inadequate training, and weak leadership commitment have been identified (Gianni and Gotzamani, 2015), but how these barriers interact with healthcare-specific challenges, such as regulatory complexity and clinical priorities, is not well understood. Furthermore, although healthcare accreditation bodies and national strategies occasionally promote asset-related practices, comprehensive AMS frameworks are rarely systematically integrated into policy or practice. This discrepancy suggests a critical misalignment between the unique operational context of healthcare and the generic AMS frameworks often borrowed from industrial sectors. Additionally, this study takes a broad organizational perspective, recognizing AMS as a systemic entity rather than as fragmented lifecycle stages. Thus, this study addresses a clear and urgent gap by developing a sector-specific understanding of AMS adoption barriers in healthcare. Using a Delphi–AHP approach, it prioritizes these barriers based on expert consensus. In doing so, the study contributes to the growing literature on asset management (Silva and Martha De Souza, 2021) by introducing a structured, hierarchical perspective based on healthcare-specific implementation challenges. By incorporating insights from healthcare professionals at various organizational levels, the study brings the discourse on asset management into a complex, high-stakes sector where AMS has been under-explored.

The study aims to contribute novel insights to the literature on asset management, particularly in the healthcare sector, where existing knowledge is fragmented. The findings will lay the groundwork for interventions and policy reforms targeted at overcoming the unique challenges of AMS implementation in healthcare organizations. This research is motivated by the need to answer the question of what hinders the implementation of AMS in healthcare. Therefore, the central research question was developed:

RQ.
What are the main barriers to the implementation of asset management systems in healthcare organizations?

To address the research question and guide the empirical inquiry, this study introduces a conceptual framework (Table 1) that categorizes potential AMS implementation barriers into five theory-driven, overarching dimensions: strategic, human resources, contextual, structural, and procedural. These categories are based on a multidisciplinary body of literature, particularly studies focusing on management system implementation, and aim to capture key facets of AMS adoption. These facets range from leadership and workforce-related issues to structural capacity and procedural alignment. Although alternative classifications are acknowledged in the literature, this framework was chosen for its conceptual coherence, thematic breadth, and demonstrated relevance to healthcare contexts. Accordingly, the framework synthesizes findings from related research domains, including asset management, quality management, and healthcare infrastructure and facilities, where similar organizational challenges have been identified. The framework serves as a theoretical foundation for the Delphi-AHP investigation and provides a lens through which to interpret barriers specific to healthcare settings. Table 1 presents the five dimensions alongside their descriptions and preliminary propositions based on existing literature.

**Table 1:** Conceptual comparison of asset management barriers across sectors

Barrier category	Industrial sector	Healthcare sector	Conceptual proposition
*Strategic*	Under-integration of asset management into strategic planning due to operational silos and short-term cost optimization focus Required shifts from traditional maintenance to maximizing asset value	Deficient leadership; lack of top management support; poor understanding of management system; unclear mission, vision, and objectives; weak accountability structures	While strategic barriers in industrial settings are often characterized by the low prioritization of AMS in favor of short-term operational objectives, healthcare organizations may encounter additional challenges. These factors may include a limited understanding of the strategic value of AMS, unclear organizational priorities, weak governance structures, and difficulties in integrating AMS into existing management and operational frameworks
*Supporting references*	Konstantakos *et al*. (2024), Nowakowski *et al.* (2017), Maletič *et al*. (2023), Beitelmal *et al*. (2017)	Johannesen and Wiig (2017), Rawshdeh *et al*. (2024), Wardhani *et al*. (2009)	
*Human Resources*	Limited internal asset management capacity, shortage of qualified personnel, and insufficient internal training and knowledge retention	Overworked clinical and technical staff; Lack of management system-specific training among clinical/technical staff; resistance to change; lack of involvement and recognition	The implementation of AMS in healthcare is likely to involve distinct human resource challenges, such as staff overload stemming from expanded responsibilities across the asset lifecycle, potentially insufficient training in strategic asset management practices, unclear role definitions that may reduce motivation, and limited involvement of staff in asset-related decision-making processes
*Supporting references*	Gomaa (2025), Konstantakos *et al*. (2024)	Kunnen *et al*. (2023), Wardhani *et al*. (2009)	
*Contextual*	Lack of cross-departmental alignment, ineffective communication, and absence of a shared asset management culture	Poor internal communication, weak teamwork, inadequate organizational culture, interpersonal conflict, fear of failure/change, lack of cooperation and trust	The dearth of a mature asset management culture is widely acknowledged as a pervasive impediment across both the industrial and healthcare sectors, though its manifestations vary. In industrial contexts, challenges frequently arise from siloed organizational structures and a predominant focus on short-term operational efficiency. Conversely, healthcare organizations may encounter supplementary relational and psychological impediments, including constrained interdisciplinary collaboration, reluctance to adopt change, and ambiguous role ownership. These factors have the potential to hinder the development and effective implementation of an AMS
*Supporting references*	Rivera *et al*. (2022), Rodríguez-Hernández *et al.* (2025), Beitelmal *et al*. (2017)	Kunnen *et al*. (2023), Wardhani *et al*. (2009)	
*Structural*	Inadequate data collection and lack of accessible asset information, limited human and techno-economic capacity to support asset management activities, ineffective or misaligned resource allocation	Operational pressures and lack of dedicated resources (time, funding, personnel), limited access to asset-related information, lack of focus on intangible assets, structural complexity	Although both healthcare and industry face barriers such as a lack of time, personnel, and funding, it is likely that the contextual factors, operational settings, and implications of these challenges differ significantly between the two domains
*Supporting references*	Konstantakos *et al*. (2024), Ihemegbulem and Baglee (2020), Beitelmal *et al*. (2017)	Kunnen *et al*. (2023), Rider *et al*. (2019), Wardhani *et al*. (2009)	
*Procedural*	Lack of a planning framework (such as SAMP), lack of systems for measuring the efficiency and effectiveness of AMS implementation and operation	High administrative complexity, integrational misalignment between healthcare procedures, healthcare-specific standards and management systems, lack of dedicated implementation and evaluation framework	It is plausible that the procedural inertia affecting AMS implementation in healthcare arises from regulatory complexity, bureaucratic rigidity, and misaligned standardization practices
*Supporting references*	Konstantakos *et al*. (2024), De Villiers (2018), Nowakowski *et al*. (2017), Maletič *et al*. (2023), Beitelmal *et al*. (2017)	Rawshdeh *et al*. (2024), Kunnen *et al*. (2023), Rathi *et al.* (2022), Salem and Elwakil (2021)	

**Source(s):** Authors’ own work

This framework is not presented as a definitive typology, but rather as a conceptual starting point for healthcare-specific research on AMS. It provides a structured lens through which to explore how known barriers – while conceptually aligned with those observed in industrial sectors – may manifest in distinct ways within the clinical, regulatory, and operational contexts of healthcare organizations. The propositions included in this study are not empirically tested hypotheses; instead, they are theoretically informed statements, developed from prior literature on asset management, quality management, and healthcare operations. Their purpose is to guide the Delphi-AHP process by framing expert discussions and supporting the evaluation of how these barriers are perceived and prioritized in the healthcare setting.

## 2. Methods

### 2.1 Delphi technique

The Delphi technique was first developed in the 1950s by the RAND Corporation in the United States. However, it was not introduced until 1963 by Dalkey and Helmer to assess intangible or uncertain variables by utilizing the knowledge and experience of a group of participants (commonly referred to as panellists, experts or respondents) through an anonymous and iterative consultation method (Dalkey and Helmer, 1963). The Delphi technique is essentially a series of successive questionnaires or rounds of controlled feedback aimed at achieving the most reliable consensus of opinion from a panel of participants (Powell, 2003). The Delphi technique can be used as an alternative to traditional meetings to avoid problems caused by strong personalities, peer pressure, and status effects (Thangaratinam and Redman, 2005). This technique is widely used in business, industry, and healthcare research, and there are many methodological versions and modifications of it (Powell, 2003). Despite its advantages and disadvantages, the Delphi method is recognized as relevant in healthcare research (Nasa *et al*., 2021). Literature recommends that the Delphi method consist of at least one questionnaire survey in three iterations. The number of rounds is largely pragmatic. The first iteration identifies general questions related to the topic to be addressed. A questionnaire with open-ended questions is distributed to participants. The responses to these questions are qualitatively evaluated by sorting and categorizing them to identify common themes. Subsequent rounds are more specific, focusing on categorizing the responses according to their importance (Thangaratinam and Redman, 2005). The Delphi technique is characterized by three features: anonymity, consensus, and iteration (Diamond *et al.*, 2014). Anonymous panellists ensure limited bias and equal treatment of views to prevent dominance of participants' opinions over others' Consensus means that an agreement is reached among participants. Iteration is one of the Delphi technique's key strengths, as it allows participants to revise their responses based on group feedback (Chalmers and Armour, 2019).

There is no fixed standard for the sample size. Generally, however, the larger the sample size, the more reliable the group judgments. According to some guidelines, Delphi methods should include at least seven participants (Chalmers and Armour, 2019) and can include up to 3,000 participants (Thangaratinam and Redman, 2005). It should be noted that, as the panel grows, a certain dropout rate between rounds is to be expected (Bardecki, 1984). The homogeneity or heterogeneity of the panel is another aspect to consider. Both types of panels have their advantages. A homogeneous group may share the characteristics necessary to reach a consensus, whereas a consensus is more difficult to achieve in a heterogeneous group (Chalmers and Armour, 2019). However, including a heterogeneous group can increase the validity of the results (Baker *et al.*, 2006).

### 2.2 The analytic hierarchy method (AHP)

The AHP method developed by Saaty (1980) is a powerful multi-criteria decision making tool that has been used in numerous environments and fields such as business (Cheng and Li, 2001), engineering (Azadeh *et al.*, 2017) and healthcare (Liberatore and Nydick, 2008). It is a method of measurement through pairwise comparisons and relies on participant judgment to derive priority scales (Saaty, 2008). The AHP develops priorities between all criteria and sub-criteria within each level of the hierarchy. The methodological process of the AHP involves several phases (Saaty, 1980): (1) building the hierarchy, (2) weighting the criteria in pairwise comparisons, (3) calculating the criteria weights, and (4) producing the final ranking.

In the AHP, a problem is structured in the form of a hierarchy. Once the hierarchy is constructed, the evaluator begins the prioritization process to determine the relative importance of the elements at each level. The ratings are made in the form of pairwise comparisons to express the dominance of one element over another. A relational rating scale with real numbers from 1 to 9 was used for the ranking (Table 2).

**Table 2:** AHP scale for pair-wise comparison

Scale	Judgment
1	Equal importance
3	Moderate importance of one over the other
5	Essential or strong importance
7	Very strong or demonstrated importance
9	Extreme or absolute importance
2, 4, 6, 8	Intermediate values between the two adjacent judgments

**Source(s):** Authors’ own compilation based on Saaty (1980)

In particular, all pairwise comparisons (pwc) are typically of the form (Goepel, 2018):


(1) pwc=(a1,a2,… anpc),(x1,x2,… xnpc)

where the integers are *ai* ∈ [ 0,1], *xi* ∈ [1, *M*], *M* = 9 and *i* = 1 … *npc*, where *npc* is the number of pairwise comparisons.


(2) $$\text{npc}=\frac{n2-n\;}{2}$$

As such, for *n* criteria the *n* x *n* decision matrix is then filled from pwc. For *ai* = 0 *xi* is considered, for *ai* = 1 the reciprocal of *xi* should be taken. In order to consolidate all participant's judgments, the geometric mean and standard deviation of all K participant's individual judgments *pwck* is calculated using the following formulas (Goepel, 2018):


(3) $$\text{Sum over }K\text{ participants }pwcx=\sum_{k=1}^{K}\cdot \ln\left({pwc}_{k}\right)$$

(4) $$\text{Square sum over }K\text{ participants }pwcx^{2}=\sum_{k=1}^{K}\cdot {\left[\ln\left({pwc}_{k}\right)\right]}^{2}$$

(5) $$\text{Geometric mean }{pwc}_{CONS}=\exp\left(\frac{pwcx}{K}\right)$$

(6) $$\text{Standard deviation }{pwc}_{SD}=\exp\left(\sqrt{\frac{{pwcx}^{2}-\frac{1}{K}\;pwcx\;\cdot pwcx}{K-1}}\right)$$

The final consolidated decision matrix is formed using the following equation:


(7) $$a_{ij}^{cons}={\left(\Pi_{k=1}^{K}aij\right)}^{\frac{1}{K}}$$

The consistency of the pairwise comparison is an important aspect that must be taken into account. It shows us whether the pairs of criteria formed are consistent or not. It is possible that the evaluators are inconsistent in their judgments due to the pairwise comparisons. In this study, an online AHP-OS tool developed by (Goepel, 2018) was used. In contrast to the calculation of the consistency ratio (CR) originally proposed by (Saaty, 2008):


(8) $$\text{CR}=\frac{\lambda -n}{\left(n-1\right)\;\cdot \;\text{RI}}$$

where λ is the maximum eigenvalue of the pairwise comparison matrix, *n* is the number of attributes, and RI is the random index, the AHP-OS tool uses the linear fit proposed by (Alonso and Lamata, 2006) to calculate the CR.


(9) $$\text{CR}=\frac{\lambda -n}{2.7699\;\cdot \;n-4.3513-n}$$

### 2.3 Proposed methodology

**Figure 1:**
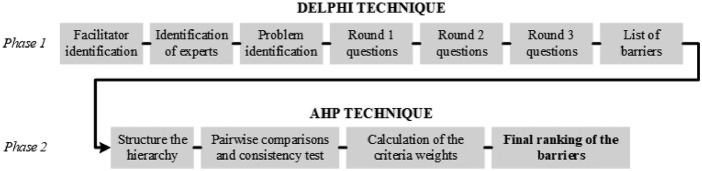
Methodological procedure used in the study. Source: Authors’ own work

The combination of the Delphi technique and the AHP is not new. The Delphi technique is typically employed to identify the most significant variables, and the AHP subsequently determines their weighting (Arof, 2015). This approach has been successful in many research areas, including addressing transportation barriers (Karam *et al.*, 2021), assessing critical success factors in construction projects (Belay *et al.*, 2021), identifying and prioritizing leadership competencies (Kim and Mallam, 2020), and evaluating the importance of digital tools or approaches in relation to hospital performance (Truong and Le, 2024). Therefore, it is argued that a combining the Delphi method with the AHP-based methodology is a proven mixed exploratory approach to investigating research phenomena in healthcare (Jiang *et al.*, 2019; Lin *et al.*, 2020). Following the research logic of the aforementioned studies, two main phases are proposed for conducting the study: (1) identification of barriers to the implementation of AMS through a participant survey (Delphi technique), (2) pairwise comparison, weighting and ranking of hierarchical criteria (AHP technique). The approach is illustrated in Figure 1. Data collection lasted approximately one month (January 2024).

#### 2.3.1 Phase 1: delphi technique

The main objective of the Delphi survey was to evaluate and prioritize the key factors that hinder the adoption of an AMS in the healthcare sector. The barrier categories were theoretically pre-defined based on prior literature, while the initial list of barriers was compiled through literature synthesis and expert consultation in the first phase of the study. The criteria for the panel were as follows: Participants must be involved in the healthcare sector (C1) and have knowledge of management systems, with a focus on AMS (C2). It should be noted that there are no current guidelines or standards for the selection of expert panel members (Shang, 2023). However, there are some generally accepted requirements for expert panel selection such as experience and knowledge, willingness and ability to participate, time to participate, and appropriate communication skills (Trevelyan and Robinson, 2015). As Delphis uses a non-random sampling technique (Keeney *et al.*, 2001), there is an inherent bias in the recruitment process as participants who are more interested in the topic are more likely to participate in the different rounds (Hasson *et al.*, 2000). To mitigate this issue, we prioritized including participants with expertise in both the clinical and managerial domains. Although experience is difficult to quantify (Nasa *et al.*, 2021), 83% of our participants had extensive professional experience, and those with less than one year were excluded. Additionally, all participants received specialized training in implementing asset management in healthcare through a structured course at the University of Maribor. Participants were selected from a pool of individuals enrolled in this course. Thirty participants consented to take part in the study. Although 90% were women and 10% were men, gender was not a selection criterion; diversity was emphasized instead in terms of professional background, institutional role, and seniority. All participants held at least a bachelor's degree and worked in various departments and at different hierarchical levels across multiple healthcare institutions. Our sample included both team leaders (20%) and team members (80%), ensuring a range of operational perspectives. Although the sample was drawn from a single training program, efforts were made to incorporate a variety of perspectives, which is critical in Delphi panels. In homogeneous panels, participants tend to share similar opinions, which can result in systematic biases in the results, either above or below the mean, rather than capturing the true distribution of views (Förster and von der Gracht, 2014). Although most participants had similar educational backgrounds in healthcare or nursing, they held diverse professional roles within their institutions. These roles ranged from frontline healthcare provider positions to managerial and leadership roles. This variation in job function, level of responsibility, years of experience, and organizational role was intended to capture a broader range of perspectives relevant to the study topic. Although the panel was selected for professional homogeneity regarding relevant expertise and training in healthcare and asset management – an approach that promotes consistency and methodological rigor – the participants also exhibited diversity in terms of job roles, institutional responsibilities, and seniority. This balance of expertise and role diversity was intended to ensure the internal validity of the consensus process and the external relevance of the findings. Furthermore, one could reasonably argue that Delphi panels composed of similarly trained and experienced experts from other fields would also yield stable and reliable results, even with a relatively small panel size, provided the participants share a common base of knowledge and understanding (Akins *et al.*, 2005).

The moderator of this study has in-depth knowledge of asset management. He is a member of the Technical Committee for Maintenance and Asset Management at the Slovenian Institute for Standardization (SIST), as well as European and international committees in the field of asset management.

Three Delphi rounds were conducted. Thirty participants took part in the first and second rounds, while 26 took part in the third, corresponding to an 86.6% retention rate. Before the first round, a face-to-face meeting was held to explain the study's purpose and methodology, as well as to encourage participants to engage in all rounds. The three online Delphi rounds were conducted using Microsoft Forms, and participants received summarized feedback after each round. The online format facilitated the participation of geographically dispersed health professionals, ensured anonymity, minimized logistical constraints, and improved data management efficiency while reducing the influence of dominant individuals on group consensus. Round 1 included open-ended questions to capture participants' thoughts on the topic under study. On average, participants generated nine ideas on potential barriers (257 in total). The facilitator reviewed all barriers. Similar barriers were merged, and duplicates and irrelevant barriers were removed. The pre-defined conceptual framework guided this refinement process, organizing the barriers into five categories: strategic, human resources, contextual, structural, and procedural. As a result, 39 unique barriers were retained. One additional barrier was proposed and included during the Delphi rounds.

Barriers were rated on a five-point Likert scale ranging from 1 (unimportant) to 5 (extremely important). A mean score of 3 was interpreted as “moderately important,” consistent with established Delphi practice (Castro-Calvo *et al.*, 2021). Consensus was defined as being achieved when ≥80% of participants rated a barrier as either “very important” or “extremely important.” Conversely, items with <20% agreement in these categories were rejected. Items falling between these thresholds were re-rated in subsequent rounds. In addition to the agreement-based threshold, an interquartile range (IQR) of ≤1 was applied as a secondary criterion for consensus. This is consistent with the recommendation that an IQR of ≤1 on a 5-point Likert scale indicates low variability and high consensus (Shang, 2023). To evaluate the stability of participant responses across rounds, we employed the McNemar chi-squared (*χ*^2^) test (Castro-Calvo *et al.*, 2021), which assesses whether changes in item ratings between rounds are statistically significant. After each Delphi round (Rounds 2 and 3), participants were given the opportunity to suggest additional barriers. One new barrier was added after Round 2. Participants also received summarized feedback from the facilitator after each round.

#### 2.3.2 Phase 2: AHP technique

An online AHP-OS tool was used, as this approach provided the participants with an easy way to make pairwise comparisons. Also, the advantage that the participants could immediately see the resulting weights and consistency index motivated us to use this approach. All participants who took part in Round 3 of the Delphi process participated in the pairwise comparison of barriers via the online AHP-OS tool. A short guide on how to use the AHP-OS tool was created and distributed to the participants. The barriers on which consensus was reached constituted the sub-criteria at Level 2 of the AHP hierarchy. The corresponding themes, which were conceptually defined based on prior literature, represented the criteria at Level 1. These thematic categories were developed *a priori* to reflect established domains relevant to the study context, rather than being derived inductively from the data. The constructed hierarchy is consistent with the recommendation of (Saaty and Ozdemir, 2003), according to which the number of items in the group should not exceed nine. Although it is expected that CR would remain low in the consolidated matrix of the larger group, we omitted two responses due to high inconsistency. Thus, 26 responses were included in the final analysis.

## 3. Results

Table 3 summarizes the results of the strategic barriers. In Round 2, four barriers were rated as “very important” or “extremely important” by ≥ 80% of the participants. No further barriers were added. The remaining barriers were re-rated in the next round. As the barriers did not receive approval from the participants, they were neither retained nor rejected (agreement ratings between 70.00 and 76.92%). The results remained stable in round three. The McNemar chi-squared test was used to assess the stability of the responses. The *p*-values for the two barriers marked in blue in Table 3 were not significant (0.6714 and 0.3914, respectively). Accordingly, we cannot claim that the responses changed significantly between rounds. This supports the decision to not include these barriers in the final list.

**Table 3:** Results of Delphi rounds 2 and 3 in relation to strategic barriers

Strategic barrier	Participant agreement (%)	Consensus
Deficient leadership	90.00% in round 2	✓
Lack of top management support	83.33% in round 2	✓
Misunderstanding of asset management by top management	83.33% in round 2	✓
Lack of clear guidelines and strategies	80.00% in round 2	✓
Lack of consistency of objectives	88.46% in round 3	✓
Lack of clear mission and vision	76.92% in round 3	-
Difficulties in defining responsibilities and accountabilities	70.00% in round 3	-

**Note(s):** Consensus for barrier inclusion was reached when ≥ 80% of participants rated the barrier as “very important” or “extremely important,” and the IQR was ≤ 1. No consensus for inclusion occurred when > 20% but < 80% of participants rated the barrier as “very important” or “extremely important.”

**Source(s):** Authors’ own work

Of the eight human resources barriers, four reached consensus in Round 2 (Table 4). Four barriers were re-rated in Round 3. In only one case did the participants reach an agreement. The results remained unchanged for the other three. The participants neither rejected nor accepted them. According to the McNemar chi-squared test results, the *p*-values for the barriers in the blue cells were not significant (0.05678, 0.5754, and 0.551, respectively). Therefore, we can conclude that there is no significant difference between rounds two and three in terms of the group's decision.

**Table 4:** Results of Delphi rounds 2 and 3 in relation to human resources barriers

Human resources barrier	Participant agreement (%)	Consensus
Overworked employees	90.00% in round 2	✓
Insufficient knowledge of employees	86.66% in round 2	✓
Lack of training and education of employees	86.66% in round 2	✓
Insufficient motivation of employees	83.33% in round 2	✓
Resistance to change	80.76% in round 3	✓
Lack of employee involvement in the decision-making process	76.92% in round 3	-
Lack of reward for success	53.84% in round 3	-
Lack of employee involvement	65.38% in round 3	-

**Note(s):** Consensus for barrier inclusion was reached when ≥ 80% of participants rated the barrier as “very important” or “extremely important,” and the IQR was ≤ 1. No consensus for inclusion occurred when > 20% but < 80% of participants rated the barrier as “very important” or “extremely important.”

**Source(s):** Authors’ own work

As shown in Table 5, participants in Round 2 could only agree on one contextual barrier. Of the six barriers re-rated in the next round, the participants accepted four, and the decision for the other two did not change. The McNemar chi-squared test showed that there was no significant difference between Rounds 2 and 3 for the latter two barriers (0.1002 and 0.8897, respectively).

**Table 5:** Results of Delphi rounds 2 and 3 in relation to contextual barriers

Contextual barrier	Participant agreement (%)	Consensus
Poor internal communication	83.33% in round 2	✓
Lack of teamwork	84.61% in round 3	✓
Inadequate organizational culture	80.76% in round 3	✓
Poor interpersonal relationships	80.76% in round 3	✓
Fear (of novelty, failure, risks)	80.76% in round 3	✓
Insufficient cooperation among staff	65.38% in round 3	-
Lack of trust between employees	61.53% in round 3	-

**Note(s):** Consensus for barrier inclusion was reached when ≥ 80% of participants rated the barrier as “very important” or “extremely important,” and the IQR was ≤ 1. No consensus for inclusion occurred when > 20% but < 80% of participants rated the barrier as “very important” or “extremely important.”

**Source(s):** Authors’ own work

Table 6 shows that the participants approved three structural barriers for inclusion in Round 2. The remaining two were re-rated in Round 3. One barrier was affirmed, one was neither affirmed nor denied, and one was rejected. The McNemar chi-squared test confirmed that there was no significant difference between the rounds (*p* = 0.6831).

**Table 6:** Results of Delphi rounds 2 and 3 in relation to structural barriers

Structural barrier	Participant agreement (%)	Consensus
Lack of qualified personnel	86.66% in round 2	✓
Lack of adequate IT infrastructure (outdated/incompatible)	83.33% in round 2	✓
Lack of time	83.33% in round 2	✓
Lack of financial support	80.76% in round 3	✓
Lack of quality data	57.69% in round 3	-

**Note(s):** Consensus for barrier inclusion was reached when ≥ 80% of participants rated the barrier as “very important” or “extremely important,” and the IQR was ≤ 1. No consensus for inclusion occurred when > 20% but < 80% of participants rated the barrier as “very important” or “extremely important.”

**Source(s):** Authors’ own work

As shown in Table 7, three procedural barriers were added in Round 2 according to the participants' agreement. One barrier was added in Round 2 and accepted by the participants in Round 3. For the other barriers, neither inclusion nor rejection was agreed upon, as in the previous round. The McNemar chi-squared test confirmed the stability of the results, with *p*-values ranging from 0.07142 to 0.6583. Therefore, no further rounds were required.

**Table 7:** Results of Delphi rounds 2 and 3 in relation to procedural barriers

Procedural barrier	Participant agreement (%)	Consensus
Difficulties in introducing new processes	86.66% in round 2	✓
Increased bureaucracy	83.33% in round 2	✓
Lack of consulting support	80.00% in round 2	✓
Time consuming effort for improvements	80.76% in round 3	✓
Misunderstanding of the benefits of AMS	76.92% in round 3	-
Misunderstandings about ISO standards	61.53% in round 3	-
Restrictions due to laws and regulations	61.53% in round 3	-
Lack of planning of AMS implementation	57.69% in round 3	-
Lack of commitment to innovation and continuous improvement	57.69% in round 3	-
Lack of understanding of stakeholder’ needs	57.69% in round 3	-
Difficulties in interpreting ISO clauses and requirements	53.84% in round 3	-
Time consuming approval procedures	53.84% in round 3	-
Unrealistic expectations of AMS	50.00% in round 3	-

**Note(s):** Consensus for barrier inclusion was reached when ≥ 80% of participants rated the barrier as “very important” or “extremely important,” and the IQR was ≤ 1. No consensus for inclusion occurred when > 20% but < 80% of participants rated the barrier as “very important” or “extremely important.”

**Source(s):** Authors’ own work

**Figure 2:**
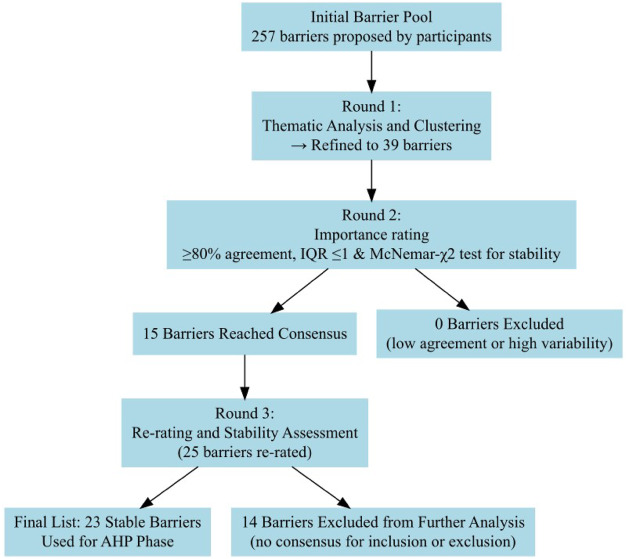
Flow diagram illustrating the filtering and consensus process across the three Delphi rounds. Source: Authors’ own work

To illustrate how the barrier list evolved across the Delphi rounds and to improve the transparency of the selection process, a flow diagram (Figure 2) was developed. This diagram outlines the progression of barriers from initial identification in Round 1 through thematic refinement, consensus assessment, and final selection for the AHP phase. In addition to applying consensus thresholds (≥80% agreement and IQR ≤1), we used the McNemar *χ*^2^ test to assess the stability of participants' responses between Round 2 and Round 3. This statistical test allowed us to assess whether the changes in ratings between rounds were significant or reflected stable judgments, further supporting the reliability of the final barrier list.

Based on the results of the Delphi study, the panellists were asked to prioritize the barriers by conducting a series of pairwise comparisons for the barriers on which consensus was reached. Table 8 shows the hierarchical structure and the results of the pairwise comparisons. Accordingly, Table 8 lists the level 1 criteria (i.e. issues related to the barriers) and the level 2 sub-criteria (i.e., barriers). The priority weights are also indicated. The priority weights are divided into “local weights” - the priority weight in relation to the previous hierarchy level — and “global weights” - the priority weight in relation to the highest hierarchy level — the goal. The global weighting is calculated by multiplying the local weights of each sub-criterion by the local weight of the corresponding main criterion. The consolidated decision matrices for the group with the corresponding group CR are shown in Appendix.

**Table 8:** The local and global weights of the five barrier categories or criteria and 23 barriers or sub-criteria

Category (local weight)	Criteria/sub-criteria	Local weights	Global weights (%)	Ranking
Strategic barrier 0.253	Deficient leadership	0.301	7.6	*3*
	Lack of top management support	0.159	4.0	11
	Misunderstanding of asset management by top management	0.166	4.2	9
	Lack of clear guidelines and strategies	0.209	5.3	6
	Lack of consistency of objectives	0.166	4.2	9
Human resources barrier 0.248	Overworked employees	0.342	8.5	*2*
	Insufficient knowledge of employees	0.196	4.9	7
	Lack of training and education of employees	0.183	4.5	8
	Insufficient motivation of employees	0.155	3.8	13
	Resistance to change	0.124	3.1	16
Contextual barrier 0.155	Poor internal communication	0.189	2.9	17
	Lack of teamwork	0.214	3.3	14
	Inadequate organizational culture	0.166	2.6	19
	Poor interpersonal relationships	0.251	3.9	12
	Fear (of novelty, failure, risks)	0.180	2.8	18
Structural barrier 0.260	Lack of qualified personnel	0.377	9.8	*1*
	Lack of adequate IT infrastructure (outdated/incompatible)	0.126	3.3	15
	Lack of time	0.223	5.8	5
	Lack of financial support	0.274	7.1	4
Procedural barrier 0.084	Difficulties in introducing new processes	0.303	2.5	20
	Increased bureaucracy	0.260	2.2	21
	Lack of consulting support	0.239	2.0	22
	Time consuming effort for improvements	0.198	1.7	23

**Source(s):** Authors’ own compilation applying the data generated by the AHP-OS tool

## 4. Discussion

### 4.1 Interpretation of findings

This study aimed to identify the barriers to implementing AMS in healthcare organizations. Given its benefits, AMS must be integrated into every organization across all businesses and sectors. Healthcare organizations must manage their assets efficiently and effectively to ensure quality and safety (Häggström *et al.*, 2023; Waring *et al.*, 2016) as well as economic viability (Beauvais *et al.*, 2019). Furthermore, healthcare assets share many characteristics with core infrastructure assets, particularly with regard to equipment and medical device investments (Grennan *et al.*, 2022) and facility management costs (Shohet and Lavy, 2004). Therefore, maintaining a high level of service in the healthcare sector and extending the life of assets is of great importance. Regardless of industry or sector, implementing AMS requires a strategic, managerial approach to asset management to optimize performance, minimize risk, and maximize value (El-Akruti *et al.*, 2013). Although responses to asset management initiatives vary, the key message is simple: effective implementation of AMS requires both management and strategic attention. Recent research shows that an organization's decision to adopt asset management is a strategic one (El-Akruti *et al.*, 2013). Understanding the rate at which AMS is adopted in a given situation requires analyzing factors that might inhibit its implementation. This study identified a wide range of issues that must be addressed for a healthcare organization to successfully implement and use AMS. This new focus, namely the barriers to AMS adoption in healthcare, is a promising area for research. Previous research has examined the importance of asset inventory in healthcare, particularly intangible assets (Rider *et al.*, 2019), or has focused narrowly on the asset management of healthcare facilities (Salem and Elwakil, 2023). However, a focus on implementing integrated, efficient management systems, such as asset management, is missing. This type of management is becoming increasingly important in all types of organizations to promote the delivery of safe, efficient, and high-quality services (El-Akruti and Dwight, 2013). Technological advancements in healthcare (Stoeva, 2020) coupled with the use of a wide range of medical devices and equipment lead to a growing need for a systematic approach, such as AMS. In this regard, the present study contributes to the literature by addressing critical factors for transitioning to an efficient AMS.

Our study showed that strategic and structural barriers are the most common. In line with one participant's response that “the implementation of the asset management system is hindered by a lack of leadership, insufficient communication, and insufficient employee involvement in decision-making, reflecting a top-down, disengaged leadership approach,” our findings underscore the critical role of strategic barriers in hindering effective system implementation. Top management commitment is paramount to successfully implementing an AMS. This process begins with a Strategic Asset Management Plan (SAMP), which outlines the role of the AMS in achieving the organization's goals and requirements (Kohl, 2020). This could be considered the starting point for aligning the organization's goals with the asset management plan. This is followed by the adequate allocation of assets and resources to implement and maintain an effective AMS (Hastings, 2015). This is consistent with previous studies that have highlighted the importance of strong leadership in supporting the implementation of AMS, e.g., in managing infrastructure assets (Beitelmal *et al.*, 2017). In the context of healthcare, effective leadership is essential for any improvement initiative or organizational change (Nawanir and Fauzi, 2024). Furthermore, understanding the purpose and intent of the AMS is essential for successfully implementing the system. This idea is supported by studies examining quality management systems in healthcare organizations (Rawshdeh *et al.*, 2024). Although culture was not deemed the most important factor (it received a global priority of 2.6%), it should not be overlooked because it is inextricably linked to leadership. Leadership certainly plays a key role in creating a culture that supports and encourages the active participation of employees at all levels (Hastings, 2015). However, differences in organizational culture exist between private organizations and the public sector (Schraeder *et al.*, 2005). Regarding AMS, previous studies have reported challenges in developing a proactive organizational culture in the public sector (Xerri *et al.*, 2015). Given the differences between the private and public sectors in healthcare, it is important to highlight the challenges organizations may face.

As previously mentioned, structural barriers are extremely important for the implementation of AMS. In this context, the lack of qualified personnel (a 9.8% global priority) and the lack of financial resources (a 7.16% global priority) are barriers that must be carefully addressed when implementing AMS in healthcare organizations. As one participant in the Delphi study noted: “Due to a shortage of staff, individuals who are not sufficiently qualified or lack the necessary knowledge and experience for certain tasks are being recruited. As a result, we are faced with a constant shortage of staff and expertise.” Since understaffing is a significant threat to many healthcare organizations (Baker *et al.*, 2019), it is expected that this barrier will be a high priority. Additionally, limited time and human resources in healthcare can lead to resistance to change, which could hinder the successful implementation of any improvement initiative or management system (Rawshdeh *et al.*, 2024). As understaffing affects the quality of care and patient safety (Senek *et al.*, 2022) and is associated with lower job satisfaction, burnout and turnover (Ausserhofer *et al.*, 2014), it should be a high priority. This is especially important when considering that employee overwork has been given a high global priority (8.5%). An increased workload can lead to occupational stress and burnout for healthcare workers (Chou *et al.*, 2014; Dobnik *et al.*, 2018). Structural barriers are similar to barriers to implementing quality management systems in healthcare (Rawshdeh *et al.*, 2024), suggesting a lack of time and financial resources could affect implementation. A lack of financial resources, for example, the organization not having the necessary budget to implement, deploy, and update the system, is also an inhibiting factor. This has been identified in asset management literature (Beitelmal *et al.*, 2017).

Overall, the identified and prioritized barriers are consistent with general challenges and barriers previously identified in asset management literature. These barriers could be associated with categories such as strategy and decision-making, managerial and organizational aspects, and resources (e.g. human and financial resources) (Beitelmal *et al.*, 2017; Maletič *et al.*, 2023). In healthcare specifically, examples of barriers mentioned in the literature related to the implementation of selected approaches and/or management systems include lack of organizational commitment, weak organizational leadership and culture, insufficient strategic direction, limited resources, lack of clear policies and procedures, lack of knowledge, poor communication, departmental silos, resistance to change, and unfamiliarity with standardization (Rawshdeh *et al.*, 2024; Shafaghat *et al.*, 2021).

### 4.2 Research implications

From a theoretical perspective, this study contributes to the literature on identifying and classifying barriers to implementing AMS in healthcare organizations. To the best of our knowledge, no previous study has examined these barriers. In summary, the study's main contribution is consolidating theoretical and practical findings on the main barriers to AMS adoption in healthcare organizations through Delphi-AHP analysis. This contribution allows researchers to explore the topic in depth and empirically confirm the relationship between barriers and AMS adoption. The managerial implications relate to lessons learned, providing a framework that delineates barriers that can facilitate and simplify healthcare organizations' transition to adopting AMS. A thorough understanding of these barriers is essential for planning and developing asset management practices and systems and can also serve as a basis for diagnosing and prioritizing AMS-related implementation actions. Accordingly, this research can provide general information to assist healthcare managers in determining an effective AMS implementation strategy, particularly in analyzing and evaluating the appropriateness of the implementation approach. Additionally, bodies that promote asset management, such as professional associations, certification bodies, and consultancies, should support healthcare organizations in overcoming barriers to AMS adoption.

Another point to consider is the fact that emerging trends such as the digital transformation of healthcare and evolving hospital accreditation requirements are increasingly overlapping with asset management practises. The integration of digital technologies (Senbekov *et al.*, 2020) – such as electronic health records (EHRs), Internet of Medical Things (IoMT) and predictive maintenance tools, facilitate or hinder the adoption of AMS. On the one hand, these technologies offer opportunities for real-time asset tracking and performance monitoring. On the other hand, they introduce new complexities in terms of data integration, cybersecurity, and staff training (Borges do Nascimento *et al.*, 2023). Similarly, accreditation frameworks often include infrastructure, asset security, and maintenance protocol criteria, indirectly influencing AMS prioritization (Gupta *et al.*, 2023). These developments underscore the evolving landscape of AMS barriers, suggesting that successful implementation necessitates alignment with overarching strategies for digital and integrated management systems (e.g. Huaytan *et al.*, 2024).

One potential social impact relates to quality and safety. Healthcare organizations that adopt AMS could refine their processes, identify inefficiencies, and implement improvements that enhance the quality and safety of their services. This could lead to greater trust and satisfaction among stakeholders. Furthermore, this study could prompt health professionals and policymakers to adopt a sector-wide asset management strategy. A key initiative in this regard is ISO 55011 (2024), which provides guidance for developing public policy to enable asset management and specifically addresses the implementation of asset management through public policy at all levels: municipal, regional, state/provincial, and national. Additionally, adopting an ISO 55011-aligned healthcare asset management roadmap offers significant national-level benefits by improving the efficiency, sustainability, and resilience of healthcare infrastructure and services. This approach provides a structured framework for managing public sector assets and creates value through lifecycle planning, risk-based decision-making, and performance optimization. At the national level, these benefits can lead to the better allocation of limited healthcare resources, lower operating costs, and greater service reliability.

### 4.3 Practical implications and recommendations

Table 9 presents the most critical barriers to AMS implementation in healthcare, prioritized by their global weights. It also presents tailored short-term interventions and long-term strategies. These interventions are designed to be actionable within the healthcare context. They aim to address immediate operational challenges and facilitate systemic transformation to ensure the sustainable adoption of AMS.

**Table 9:** Prioritized barriers by category with practical implications

Category	Barrier	Global weight (%)	Rank	Short-term interventions	Long-term strategies
Structural	Lack of qualified personnel	9.8	1	Conduct AMS-specific skill audits and reallocate non-clinical asset tasks to technical and support roles	Establish dedicated AMS roles and collaborate with educational institutions to develop AMS certification and training pathways
Human Resources	Overworked employees	8.5	2	Reassign administrative duties from the clinical staff to the AMS to clarify roles and reduce overload	Integrate AMS into human resource planning and hire or train dedicated AMS coordinators
Strategic	Deficient leadership	7.6	3	Brief senior leaders on the impact of AMS using data on risk, cost, and compliance	Include AMS in executive performance KPIs and embed it into institutional strategic plans
Structural	Lack of financial support	7.1	4	Highlight the ROI of lifecycle asset decisions and identify short-term savings	Allocate dedicated AMS budgets and include AMS in long-term capital investment strategies
Structural	Lack of time	5.8	5	Prioritize AMS activities with the highest operational value and reduce duplicative documentation	Schedule dedicated AMS review cycles and integrate AMS planning into daily workflows
Strategic	Lack of clear guidelines and strategies	5.3	6	Co-develop interim AMS guidelines that are adapted to the local context	Develop and implement strategic asset management plans (SAMPs) that align with ISO 55000 standards
Human Resources	Insufficient knowledge of employees	4.9	7	Conduct an introductory training session on AMS concepts and lifecycle value	Integrate AMS topics into continuous professional development and clinical training programs
Human Resources	Lack of training and education of employees	4.5	8	Hold sessions that focus on the asset lifecycle, maintenance planning, and ISO standards	Incorporate AMS training into orientation and continuing medical education (CME) programs
Strategic	Misunderstanding of asset management by top management	4.2	9 (tie)	Use case studies and analogies relevant to healthcare to illustrate AMS principles	Include AMS in leadership development programs and create executive awareness initiatives for AMS
Strategic	Lack of consistency of objectives	4.2	9 (tie)	Facilitate strategic alignment workshops for different departments	Incorporate AMS goals into organizational performance frameworks and strategy maps

**Source(s):** Authors’ own work

Key barriers, such as a lack of qualified personnel and overworked employees, highlight urgent workforce capacity issues. These issues call for rapid task reallocation and the longer-term development of specialized AMS roles and educational partnerships. Strategic barriers, such as deficient leadership and a lack of clear guidelines, underscore the need to engage top management through targeted briefings and to embed AMS objectives into institutional performance and planning frameworks. The existence of structural limitations, including but not limited to financial support and time constraints, necessitates the advocacy for dedicated resources and the integration of AMS activities into routine workflows.

The identified barriers and prioritized interventions lay the groundwork for tailoring AMS implementation models and readiness assessments to different healthcare settings. In public institutions, where financial and bureaucratic challenges are common, readiness efforts should focus on securing sustainable funding and streamlining administrative processes. While private providers often exhibit stronger leadership, they may benefit from enhanced staff training and leadership development to overcome workforce resistance.

The relevance and intensity of barriers can vary in different institutional contexts. For instance, public sector facilities may encounter structural limitations related to centralized procurement and rigid budgeting cycles, whereas private providers may have fewer bureaucratic obstacles but may lack standardized AMS training. Similarly, healthcare facilities in rural areas often face major staff shortages and infrastructural limitations, further increasing personnel and procedural hurdles. Digital tools, such as AI-powered asset tracking and remote training, can alleviate these constraints and improve the effectiveness of AMS in such settings. Urban facilities may have difficulty disseminating AMS to larger systems. These findings can inform context-specific readiness assessments, which prioritize barriers based on the operational realities of different healthcare environments.

Furthermore, one could argue that overcoming context-specific barriers, such as implementing digital solutions in rural hospitals or holding leadership workshops for private urban providers, can significantly improve AMS adoption. Overall, integrating emerging digital technologies has the potential to overcome key barriers such as limited time and poor communication, thereby supporting the sustainable implementation of AMS across varied healthcare environments.

In summary, healthcare organizations can improve readiness, foster engagement, and accelerate the effective implementation of AMS by adopting the suggested multi-level recommendations and adapting solutions to their unique institutional and contextual realities.

### 4.4 Limitations and future research

It is imperative to acknowledge the limitations of this study. Firstly, the use of a non-random, purposive sample drawn from a single training group in Slovenia may limit the generalizability of the findings to other healthcare contexts or geographical regions. While the participants represented a variety of professional backgrounds and experience levels, it is essential to ensure validation across different healthcare settings, countries, and organizational types.

Secondly, while the Delphi–AHP methodology is effective in identifying and prioritizing barriers, it relies on expert opinion and consensus, which may introduce subjective biases. Subsequent studies could augment this approach with qualitative case studies to provide richer contextual insights and quantitative cross-sectional analyses to test the relationships between the identified barriers and AMS adoption outcomes.

Furthermore, the present study primarily concentrated on the identification and prioritization of barriers, without directly assessing organizational readiness or the success of implementation. It is thus recommended that subsequent studies focus on the development and validation of AMS readiness assessment tools that are tailored to the healthcare sector. These tools have the potential to assist institutions in identifying their particular challenges and capacities prior to the adoption of AMS.

Despite the absence of a direct comparison between healthcare settings in the present study, extant literature and practical considerations suggest that AMS implementation barriers may significantly differ between public and private organizations, as well as between urban and rural institutions. These differences can be attributed to variations in funding structures, governance models, and resource availability. It is advised that subsequent research endeavor to investigate these distinctions in order to inform the development of tailored AMS implementation strategies.

Finally, given the increasing digitization of healthcare asset management, future studies should examine how emerging digital tools, such as artificial intelligence, predictive maintenance, and automated workflow systems, can mitigate key barriers like insufficient time, poor communication, and limited data accessibility. Understanding how technology can improve AMS adoption is essential to advancing effective, sustainable asset management in healthcare.

## 5. Conclusion

In the face of mounting cost pressures and regulatory demands, AMSs provide healthcare organizations with a structured approach to mitigating risk and aligning assets with strategic objectives. This study identifies and prioritizes the main barriers to AMS implementation from the perspective of healthcare professionals. This study offers novel insights into the relatively under-explored domain of healthcare management literature. The prevailing challenges encompass inadequate leadership commitment, insufficient time and qualified personnel, and employee overload.

Utilizing a combined Delphi–AHP approach, this study methodically identifies and categorizes barriers to AMS adoption. The findings emphasize the critical need for targeted interventions that address strategic, human resource, structural, and procedural challenges to enhance AMS uptake.

Given the variability inherent in organizational contexts – differences between the public and private sectors, as well as between urban and rural settings, for example – the necessity of tailoring AMS implementation strategies has become increasingly apparent. This study establishes the foundation for developing context-sensitive implementation models, which, in turn, will support more effective AMS adoption in diverse healthcare environments.

## Data Availability

The data that support the findings of this study are available by contacting the corresponding author.

## Appendix

**Table A1:** Consolidated decision matrix of the main criteria

	I	II	III	IV	V
Strategic barrier (I)	1	1.080276	0.969756	1.645629	2.807724
Structural barrier (II)	0.925689	1	1.115276	1.69382	3.205259
Human resources barrier (III)	1.031188	0.896639	1	1.751124	2.721324
Contextual barrier (IV)	0.60767	0.590381	0.571062	1	2.071967
Procedural barrier (V)	0.35616	0.311987	0.367468	0.482633	1

**Note(s):** CR: 0.002306

**Table A2:** Consolidated decision matrix of the strategic barrier sub-criterion

	I	II	III	IV	V
Lack of top management support (I)	1	0.622116	0.647958	1.057311	0.851037
Deficient leadership (II)	1.607418	1	1.803034	1.812241	1.686977
Lack of clear guidelines and strategies (III)	1.54331	0.554621	1	1.311861	1.277946
Misunderstanding of asset management by top management (IV)	0.945795	0.551803	0.762276	1	1.158464
Lack of consistency of objectives (V)	1.175037	0.592776	0.782505	0.863212	1

**Note(s):** CR: 0.006925

**Table A3:** Consolidated decision matrix of the human resources barrier sub-criterion

	I	II	III	IV	V
Insufficient motivation of employees (I)	1	0.888924	0.766529	1.30322	0.429393
Insufficient knowledge of employees (II)	1.124955	1	1.282051	1.573322	0.53312
Lack of training and education of employees (III)	1.304582	0.78	1	1.676487	0.512373
Resistance to change (IV)	0.76733	0.635598	0.596485	1	0.426637
Overworked employees (V)	2.328868	1.875751	1.951704	2.343915	1

**Note(s):** CR: 0.004921

**Table A4:** Consolidated decision matrix of the contextual barrier sub-criterion

	I	II	III	IV	V
Inadequate organizational culture (I)	1	0.886537	0.687072	0.709476	0.968242
Poor internal communication (II)	1.127984	1	0.738587	0.932628	1.035935
Poor interpersonal relationships (III)	1.455451	1.353937	1	1.317388	1.258013
Lack of teamwork (IV)	1.409491	1.072239	0.759078	1	1.270198
Fear (of novelty, failure, risks) (V)	1.0328	0.965312	0.794904	0.787279	1

**Note(s):** CR: 0.001949

**Table A5:** Consolidated decision matrix of the structural barrier sub-criterion

	I	II	III	IV
Lack of qualified personnel (I)	1	1.897036	1.370264	2.701651
Lack of time (II)	0.527138	1	0.968563	1.665445
Lack of financial support (III)	0.729786	1.032457	1	2.545686
Lack of adequate IT infrastructure (outdated/incompatible) (IV)	0.370144	0.60044	0.392821	1

**Note(s):** CR: 0.007573

**Table A6:** Consolidated decision matrix of the procedural barrier sub-criterion

	I	II	III	IV
Difficulties in introducing new processes (I)	1	1.27169	1.640877	1.085731
Increased bureaucracy (II)	0.786355	1	1.450792	1.090305
Time consuming effort for improvements (III)	0.60943	0.689279	1	0.979275
Lack of consulting support (IV)	0.921039	0.917174	1.021164	1

**Note(s):** CR: 0.006827

**Source(s):** Authors’ compilation
